# Gene-carbohydrate and gene-fiber interactions and type 2 diabetes in diverse populations from the National Health and Nutrition Examination Surveys (NHANES) as part of the Epidemiologic Architecture for Genes Linked to Environment (EAGLE) study

**DOI:** 10.1186/1471-2156-15-69

**Published:** 2014-06-14

**Authors:** Raquel Villegas, Robert J Goodloe, Bob E McClellan, Jonathan Boston, Dana C Crawford

**Affiliations:** 1 Vanderbilt Epidemiology Center, Department of Medicine, Vanderbilt University Medical Center, 2525 West End Avenue, Suite 600, Nashville, TN 37203-1738, USA; 2 Molecular Physiology and Biophysics, 1211 Medical Center Drive, Nashville, TN 37232, USA; 3 Center for Human Genetics Research, 519 Light Hall Vanderbilt University Medical Center, 2215 Garland Avenue, Nashville, TN 37232-0700, USA

**Keywords:** Type 2 diabetes, Carbohydrate, Fiber, Gene

## Abstract

**Background:**

Both environmental and genetic factors impact type 2 diabetes (T2D). To identify such modifiers, we genotyped 15 T2D-associated variants from genome-wide association studies (GWAS) in 6,414 non-Hispanic whites, 3,073 non-Hispanic blacks, and 3,633 Mexican American participants from the National Health and Nutrition Examination Surveys (NHANES) and evaluated interactions between these variants and carbohydrate intake and fiber intake.

**Results:**

We calculated a genetic risk score (GRS) with the 15 SNPs. The odds ratio for T2D with each GRS point was 1.10 (95% CI: 1.05-1.14) for non-Hispanic whites, 1.07 (95% CI: 1.02-1.13) for non-Hispanic blacks, and 1.11 (95% CI: 1.06-1.17) for Mexican Americans. We identified two gene-carbohydrate interactions (*P* < 0.05) in non-Hispanic whites (with *CDKAL1* rs471253 and *FTO* rs8050136), two in non-Hispanic blacks (with *IGFBP2* rs4402960 and *THADA* rs7578597), and two in Mexican Americans (with *NOTCH2* rs1092398 and *TSPAN8-LGRS* rs7961581). We found three gene-fiber interactions in non-Hispanic whites (with *ADAMT59* rs4607103, *CDKN2A/2B* rs1801282, and *FTO* rs8050136), two in non-Hispanic blacks (with *ADAMT59* rs4607103 and *THADA* rs7578597), and two in Mexican Americans (with *THADA* rs7578597 and *TSPAN8-LGRS* rs796158) at the *P* < 0.05 level. Interactions between the GRS and nutrients failed to reach significance in all the racial/ethnic groups.

**Conclusion:**

Our results suggest that dietary carbohydrates and fiber may modify T2D-associated variants, highlighting the importance of dietary nutrients in predicting T2D risk.

## Background

For the past few years, genome-wide association studies (GWAS) have identified hundreds of common variants associated with human diseases and traits, including type 2 diabetes (T2D). More than 40 genetic susceptibility loci have been reported for T2D and most reported variants have small to moderate effects and account for only a small proportion of the heritability of T2D [1]. Both environmental and genetic factors impact T2D. However, despite evidence that genetic variants and environmental factors are associated with T2D, relatively few studies have been published investigating the interaction between the two.

Most of the loci identified to date by GWAS of T2D appear to be involved in beta-cell function rather than insulin resistance [2]. GWAS of T2D-associated quantitative traits have also found more variants associated with insulin secretion rather than insulin resistance [3]. Insulin is secreted in response to elevated blood glucose concentration; thus, dietary carbohydrates, which influence glucose concentration and insulin demands [4], may modify associations between GWAS variants and T2D. Fiber, on the other hand, may influence the amount or rate of carbohydrate absorbed and, thus, may modify associations between GWAS variants and T2D. Fiber has been associated with lower risk of T2D [5,6].

In this study, we investigated associations between T2D and genetic variants discovered by GWAS in three racial/ethnic groups and modifications of these associations by carbohydrate and fiber intake. We used data from the National Health and Nutrition Examination Surveys (NHANES) as accessed by the Epidemiologic Architecture for Genes Linked to Environment (EAGLE), a study site of the larger Population Architecture using Genomics and Epidemiology (PAGE) I study established in 2008 to characterize GWAS-identified variants in diverse population-based studies [7]. We present here an investigation of the effects of 15 T2D-associated SNPs and T2D interactions with carbohydrate and fiber intake among non-Hispanic whites, non-Hispanic blacks, and Mexican Americans. We also evaluated the combined effect of genetic variants by calculating a genetic risk score (GRS) and examined possible interactions of the GRS with carbohydrate intake and fiber across the three racial/ethnic groups.

## Results

Table 1 displays descriptive statistics for the key variables in this study. On average, non-Hispanic whites were older than non-Hispanic blacks and Mexican Americans. Non-Hispanic whites and Mexican Americans had higher mean carbohydrate intake levels (264.72 and 265.06 g/day) compared with non-Hispanic blacks (255.81 g/day). Non-Hispanic blacks had lower mean fiber intake (13.46 g/day) compared with Mexican-Americans (19.48) and non-Hispanic whites (16.63 g/day). The average BMI for all groups was in the overweight category (>25 kg/m^2^), and non-Hispanic whites had the lowest average BMI of the three groups.

**Table 1:** Descriptive statistics for adult NHANES participants by population

	**Non-Hispanic whites (n = 6,414)**	**Non-Hispanic blacks (n = 3,073)**	**Mexican Americans (n = 3,633)**
**% female**	55%	56%	51%
**Mean age in years (±SD)**	52.77 (19.83)	43.74 (17.29)	43.56 (17.70)
**Age range in years**	18-90	18-90	18-90
**Mean BMI, kg/m**^**2**^**(±SD)**	27.38 (5.80)	28.87 (7.00)	28.16 (5.54)
**BMI range, kg/m**^**2**^	11.7-64.5	13.3-79.6	15-59.7
**Mean fiber in grams (±SD)**	16.63 (10.28)	13.46 (9.53)	19.48 (12.70)
**Fiber range in grams**	0.0-145.35	0.0-128.30	0.0-111.79
**Mean CH in grams (±SD)**	264.72 (128.05)	255.81 (143.32)	265.06 (130.95)
**CH range in grams**	2.8-1700.37	12.5-1330.9	2.66-1189.70

*Abbreviations:* carbohydrates (CH), body mass index (BMI), standard deviation (SD).

We tested 15 previously identified T2D SNPs for associations with T2D in NHANES data (Additional file 1: Table S1). Analyses were adjusted for BMI, age, and sex and were stratified by race/ethnicity. Of the 15 SNPs tested, only 5 (33%) were associated with T2D in any one racial/ethnic group at *P* < 0.05. As expected, the most associations, four SNPs, were observed among non-Hispanic whites (*CDKN2A/B* rs10811661, *SLC30A8* rs13266634, *IGFBP2* rs4402960, and *TCF7L2* rs7903146). Among non-Hispanic blacks and Mexican Americans, only two SNPs were associated with T2D in each population. Only one of the variants, *TCF7L2* rs7903146, was associated with T2D in all three racial/ethnic groups at *P* < 0.05. After applying the Bonferroni correction for multiple testing, the significant *P* value threshold was 0.001. *TCF7L2* rs7903146 in non-Hispanic blacks and, *IGFBP2* rs4402960 in non-Hispanic whites, had a *P* value of <0.001 (Additional file 1: Table S2).

We then calculated a genetic risk score (GRS) based on all 15 SNPs. Risk alleles were defined by previously reported T2D GWAS results for all variants (Table 2). As expected, higher genetic risk scores were significantly associated with higher risk of T2D in models adjusted for age, sex, and BMI for all three racial/ethnic groups. The odds ratio for T2D for each GRS point (per risk allele) was 1.10 (95% CI: 1.05-1.14) for non-Hispanic whites, 1.07 (95% CI: 1.02-1.13) for non-Hispanic blacks, and 1.11 (95% CI: 1.06-1.17) for Mexican Americans. The OR for quintiles of the GRS were 1.00, 0.90, 1.36, 1.36 and 1.70 (p < 0.0001) for non-Hispanic whites, 1.00, 1.07, 1.30, 1.42 and 1.70 (p = 0.0004) for non-Hispanic blacks and 1.00, 1.21, 1.16, 1.58 and 1.80 (p < 0.0001) for Mexican Americans (Table 2).

**Table 2:** Association between GRS and T2D*

	**Non-Hispanic whites**		**Non-Hispanic blacks**		**Mexican Americans**	
	**OR (95% CI)**	***P*** **value**	**OR (95% CI)**	***P*** **value**	**OR (95% CI)**	***P*** **value**
**GRS continuous**	1.1 (1.05-1.14)	0.0001	1.07 (1.02-1.13)	0.0107	1.11 (1.06-1.17)	0.0001
**Quintiles**						
**Q1**	1.00	<0.0001	1.00	0.004	1.00	<0.0001
**Q2**	1.13 (0.83-1.55)		1.07 (0.69-1.65)		1.21 (0.86-1.72)	
**Q3**	0.90 (0.65-1.24)		1.30 (0.88-1.91)		1.16 (0.83-1.62)	
**Q4**	1.36 (1.05-1.77)		1.42 (0.99-2.04)		1.58 (1.12-2.22)	
**Q5**	1.70 (1.26-2.29)		1.70 (1.13-2.56)		1.80 (1.33-2.44)	

*Adjusted for age, sex, and BMI.

### Dietary modifiers

**Figure 1:**
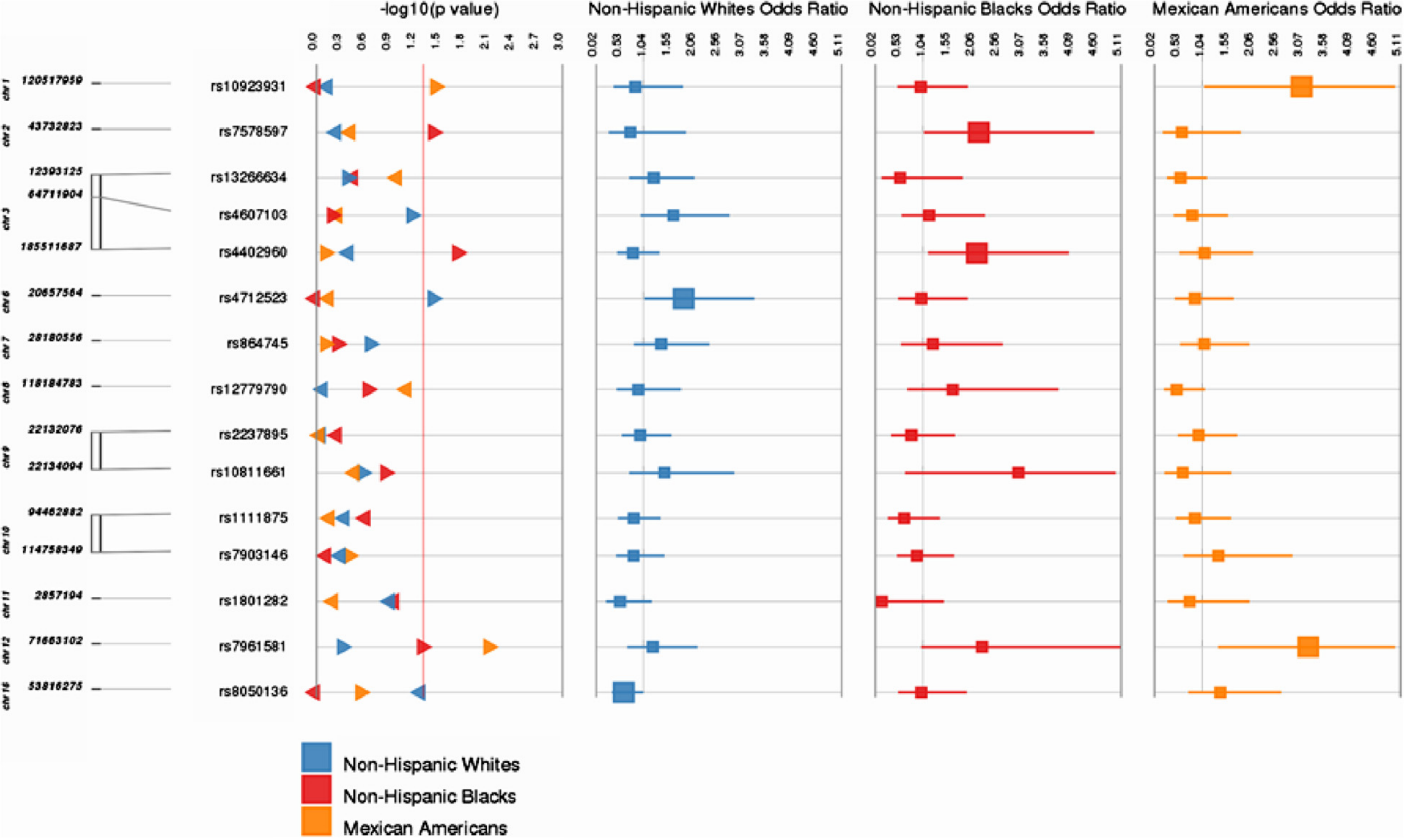
**Interaction between carbohydrates and GWAS SNPS and T2D: the potential modifying effects of carbohydrate intake on the genetic associations for T2D are shown in this figure.** OR and 95% CI for the interaction between each SNP and fiber are shown separately in the three racial/ethnic groups in 3 different columns and colors.

**Figure 2:**
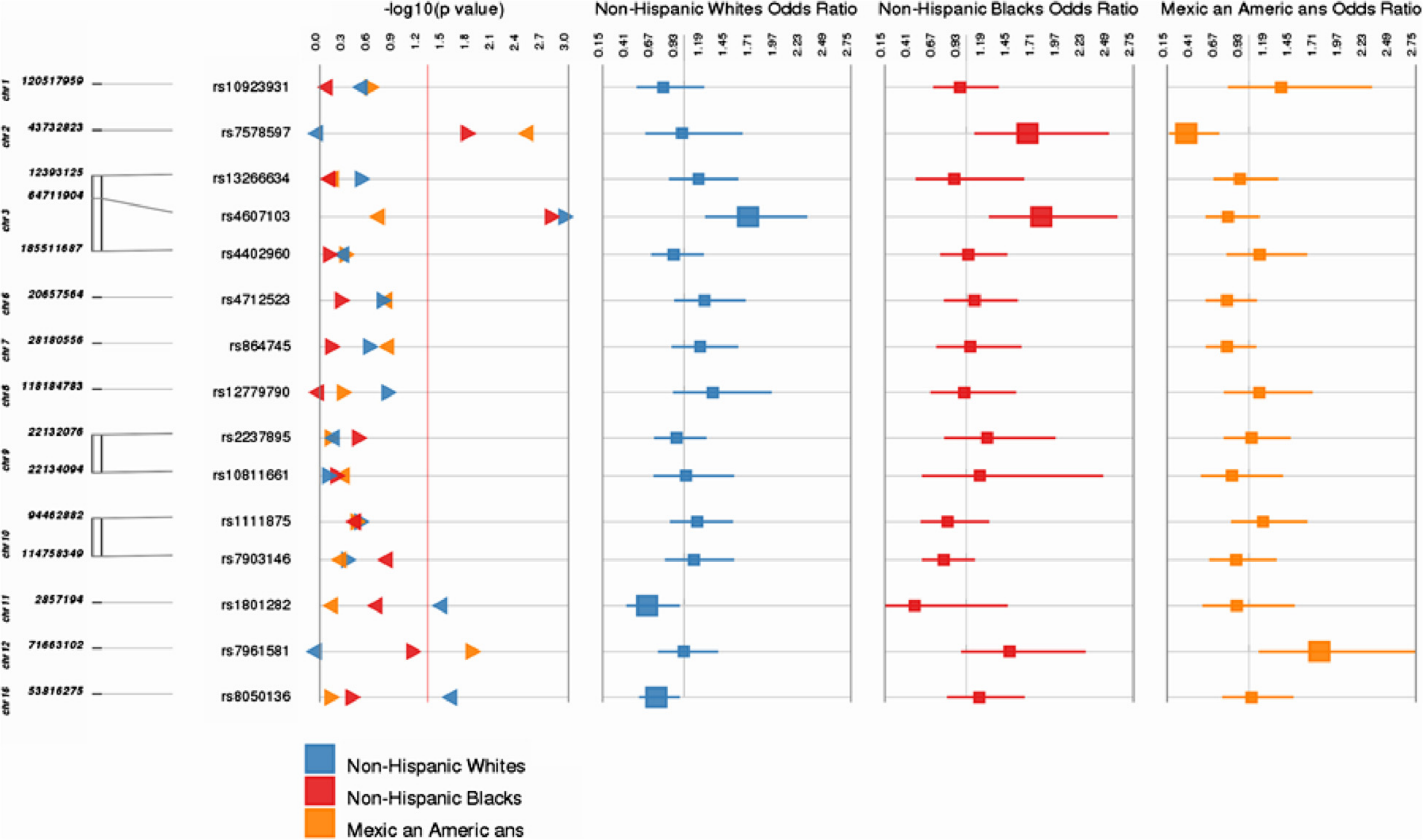
**Interaction between fiber and GWAS SNPS and T2D: the potential modifying effects of fiber intake on the genetic associations for T2D are shown in this figure.** OR and 95% CI for these interactions between SNPs and fiber are shown separately in the three racial/ethnic groups in 3 different columns and colors.

We tested for potential modifying effects of carbohydrate and fiber intake on genetic associations with T2D. To do this, we performed formal tests of interaction for all 15 SNPs and each dietary variable (Figures 1 and 2 and Additional file 1: Tables S3 and S4). We identified several gene-environment interactions at a significance threshold of *P* < 0.05. Among non-Hispanic whites, we identified two SNP × carbohydrate interactions [*CDKAL1* rs471253 (OR = 1.83; 95% CI: 1.02-3.33) and *FTO* rs8050136; (OR = 0.59; 95% CI: 0.35-1.00)] and three gene-fiber interactions [*ADAMT59* rs4607103 (OR = 1.67; 95% CI: 1.22-2.29), *CDKN2A/2B* rs1801282 (OR = 0.62; 95% CI: 0.4-0.95) and *FTO* rs8050136(OR = 0.71; 95% CI: 0.53-0.95)]. Among non-Hispanic blacks, we identified two carbohydrate-gene interactions [*IGFBP2* rs4402960 (OR = 2.12; 95% CI: 1.12-4.04) and *THADA* rs7578597 (OR = 2.16; 95% CI: 1.03-4.55)] and two gene-fiber interactions [*ADAMT59* rs4607103 (OR = 1.79; 95% CI: 1.24-2.58) and *THADA* rs7578597 (OR = 1.64; 95% CI: 1.08-2.49)]. Among Mexican Americans we identified two gene-carbohydrate interactions [*NOTCH2* rs1092398 (OR = 3.06; 95% CI: 1.05-8.93) and *TSPAN8-LGRS* rs7961581 (OR = 3.21; 95% CI: 1.34-7.7)] and two gene-fiber interactions [*THADA* rs7578597 (OR = 0.34; 95% CI: 0.17-0.69) and *TSPAN8-LGRS* rs7961581 (OR = 1.74; 95% CI: 1.1-2.75)]. Interestingly, the *ADAMT59* rs4607103-fiber interaction was observed among both non-Hispanic whites and non-Hispanic blacks with consistent directions of effect for the interaction term. Likewise, the *THADA* rs7578597-fiber interaction term was observed among both non-Hispanic blacks and Mexican Americans albeit with opposing directions of effect for the interaction term. No interaction terms for carbohydrates or fiber were significant across all three racial/ethnic groups. The *P* value after Bonferroni correction for multiple testing was 0.0005, and none of the *P* values for interaction passed multiple testing (Additional file 1: Tables S3 and S4).

Finally, we tested for interactions of the GRS with carbohydrate and fiber intakes across all racial/ethnic groups. No interactions of the GRS with carbohydrate or fiber intake and T2D were found for any racial/ethnic group. While no interaction terms in any racial/ethnic group for fiber or carbohydrate intake were significant at *P* < 0.05, the interaction term for fiber and GRS among non-Hispanic whites was of marginal significance (*P* = 0.09; Table 3).

**Table 3:** Association between interaction factors for genetic risk scores with nutrients and T2D*

	**Non-Hispanic whites**		**Non-Hispanic blacks**		**Mexican Americans**	
	**OR (95% CI)**	***P*** **value**	**OR (95% CI)**	***P*** **value**	**OR (95% CI)**	***P*** **value**
**GRS with carbohydrates**	0.95 (0.82-1.11)	0.53	1.1 (0.9-1.33)	0.36	0.93 (0.77-1.12)	0.45
**GRS with fiber**	1.07 (0.99-1.16)	0.09	1.06 (0.96-1.18)	0.25	1.05 (0.96-1.15)	0.32

*Adjusted for age, sex, BMI, kcal/day, GRS, and nutrients.

## Discussion

In this study, we investigated interactions of carbohydrate and fiber intakes with GWAS-identified genetic loci for T2D among three racial/ethnic groups in a representative sample of the US population. We examined possible interactions for both individual SNPs, as well as combinations of SNPs by calculating a GRS.

Overall, only one SNP of the 15 tested, rs7903146 in *TCFL2*, was associated with T2D in all three racial/ethnic groups. This is one of the strongest and most replicated loci associated with T2D. *TCF7L2* gene variants have been associated with impaired pancreatic beta cell function [8]. While no other associations were significant across all three racial/ethnic groups, *SLC30A8* rs13266634 was associated with T2D among both non-Hispanic whites and Mexican Americans. The *SLC30A8* gene encodes a pancreatic islet-specific Zn transporter involved in insulin biosynthesis, maturation, and storage of pancreatic beta cells and is associated with decreased insulin secretion [9]. In addition, as expected [10,11], while most SNPs were not associated with T2D in any one racial/ethnic group, genetic risk scores calculated using all 15 previously-associated SNPs were associated with T2D for each racial/ethnic group. Some data are available on GRS and T2D risk in European-ancestry populations and results were similar to ours. In a large, nested case–control study of 2,809 T2D cases and 3,501 controls from the Health Professionals Follow-up Study and Nurses’ Health Study (all of European ancestry), the GRS was calculated with 10 SNPs in 9 loci. The odds ratio for T2D for each GRS point, corresponding to 1 risk allele, was 1.19 (95% CI, 1.14 -1.24) for men and 1.16 (CI, 1.12 to 1.20) for women. In a recent study involving an African American population, the trend of increase in risk for T2D with increasing risk allele load was similar to observations in European-ancestry populations [10].

We found suggestive evidence that genetic risk for T2D is modified by the dietary components carbohydrates and fiber. More specifically, we found that carbohydrate intake modified the association between six SNPs and T2D: *CDKAL1* rs4712523 and *FTO* rs8050136 in non-Hispanic whites, *IGFBP2* rs4402960 and *THADA* rs7578597 in non-Hispanic blacks, and *NOTCH* rs10923931 and *TSPAN8-LGRS* rs7961581 in Mexican Americans. Fiber modified the association between *FTO* rs8050136 in non-Hispanic whites, *TSPAN8-LGRS* rs7961581 in Mexican Americans, and *CDKN2A/B* rs10811661 in non-Hispanic blacks. No significant interaction term was observed consistently across all three racial/ethnic groups. However, the association between two other SNPs and T2D was modified by fiber intake in two racial/ethnic groups: *ADAMTS9* rs4607103 in non-Hispanic whites and non-Hispanic blacks and *THADA* rs7578597 in non-Hispanic blacks and Mexican Americans. As noted above, the directions of effect were consistent for *ADAMTS9* rs4607103, but were in the opposite direction for *THADA* rs7578597. One possible explanation for the opposite direction of the interaction of this SNP with fiber in these two racial/ethnic groups could be that the SNP is tagging two different variants in each racial/ethnic group [12]. While these data suggest that carbohydrate and fiber intake may modify genotype-phenotype associations and that these associations may differ by race/ethnicity, further studies conducted with larger sample sizes are needed to confirm this finding.

Indeed, a major limitation of this study is sample size. While overall a large number of NHANES participants have available DNA samples (now >19,000 samples with the inclusion of NHANES 2007–2008), stratification by race/ethnicity and T2D status reduced the sample size for our analyses considerably. The stratification problem was further compounded in the gene-environment interaction analyses. Power calculations suggest that we did not have enough power to detect interactions between these variants and nutrient intakes. For example, we found an interaction for both carbohydrate intake and fiber intake with *FTO* rs8050136 among non-Hispanic whites. For the same SNP, we had sufficient power (at least 80%) to detect an interaction with carbohydrate or fiber intakes among Mexican Americans (MAF = 0.25) with an effect size of at least 1.25. For non-Hispanic blacks (MAF = 0.44), we had a smaller sample and sufficient power to detect an interaction with an effect size of at least of 1.30. These power calculations, however, may be inflated given that it is likely we did not directly genotype the causal or risk variants for non-Hispanic blacks and Mexican Americans.

Data on interactions of genes with carbohydrate or fiber intake and T2D risk are limited and most studies have examined interactions between variants in *TCFL2* (the strongest T2D locus identified to date) and carbohydrate and fiber intakes among populations of European ancestry. In the Nurse’s Health Study, interactions between the *TCFL2* (rs7903146) and glycemic load and glycemic index and the risk of T2D were found, although the risk of T2D associated with this variant did not significantly differ by cereal fiber or carbohydrate intake [4]. In the Malmo Diet and Cancer Study, interactions between *TCFL2* (rs7903146) and dietary fiber intake were found, but no association between this SNP and carbohydrate intake and T2D risk was found [13]. Interactions between two other loci that were not part of our study with carbohydrate intake and the risk of T2D have been investigated in a European-ancestry population [14,15]. This study reported a significant interaction between a common genetic variant in the glucose-dependent insulinotropic polypeptide receptor gene (*GIPR*), quintiles of carbohydrate intake (*P* = 0.0005), and incident type 2 diabetes [14] with a sex-specific interaction between a variant in the insulin receptor substrate 1 gene (rs2943641), intake of carbohydrates, and incident type 2 diabetes [15]. Interactions between wholegrain foods, a good source of fiber, and variants in this locus and the risk of T2D were found in the European Prospective Investigation into Cancer and Nutrition (EPIC)-Potsdam cohort [16]. In a large meta-analysis of 14 cohorts of European populations, interactions between several GWAS polymorphisms associated with glucose, insulin, and wholegrain foods were investigated for interactions with fasting glucose and insulin. No interaction was found between the variant in *TCFL2,* wholegrain foods, and fasting glucose, although an interaction with wholegrain food intake and rs780094 (*GCKR*) for fasting insulin (*P* = 0.006) was found [17]. An interaction between carbohydrate intake and a common genetic variant (rs10423928) in the glucose-dependent insulinotropic polypeptide receptor gene (*GIPR*), quintiles of carbohydrate intake (*P* = 0.0005), and incident T2D was observed in another study [14]. The SNP *IRS1* rs2943641 also interacted with carbohydrate intake and incident T2D in a sex-specific fashion in the Malmo Diet and Cancer Cohort [15]. Similarly, data on interactions between the GRS and dietary factors with T2D are scarce [18].

In interpreting our findings, one should consider several limitations to this study. First, NHANES is a cross-sectional study and, therefore, we were unable to determine the temporal sequence of our results. As the number of factors under study increases, as with the addition of interaction terms, so do the number of strata. With a set sample size, increasing the number of terms in the model quickly increases the degrees of freedom and reduces the per-stratum sample size, thus decreasing statistical power. For this reason, even with the relatively large sample sizes in NHANES, we had to restrict our analyses to SNPs with minor allele frequencies >5%. After using the Bonferroni correction for multiple testing, none of the *P* values for the gene-nutrient interactions met the significance threshold. Although the Bonferroni method is very conservative, it is also easier to interpret. Correcting for multiple testing in gene-environment interaction studies is inherently more complicated than in standard single-SNP association studies.

While the power of the study was limited, a major strength of the study is the fact that we had dietary data available. Most GWAS studies do not have dietary data available and, thus, cannot investigate dietary modification of genetic risk factors and T2D. The differences in T2D between individuals and between populations may partly result from the interaction of genetic variants with dietary modifiers. Thus, understanding the mechanisms behind interactions between T2D-related genetic variants and environmental factors is of critical importance to determining the etiology of T2D.

## Conclusion

Differences in T2D between individuals and between populations may partly result from interactions of known genetic variants with carbohydrate and fiber intake. The results presented here suggest that carbohydrate and fiber intake may modify the association between GWAS variants and T2D.

## Methods

### Study population

Study samples were drawn from three National Health and Nutrition Examination Surveys (NHANES III, NHANES 1999–2000, and NHANES 2001–2002) conducted by the National Center for Health Statistics (NCHS) at the Centers for Disease Control and Prevention (CDC). Participant ascertainment and data collection for NHANES have been previously described [19,20]. Race/ethnicity was self-described. Body mass index (BMI) was calculated from height and weight measured in the Mobile Examination Center by CDC medical personnel. All procedures were approved by the CDC Ethics Review Board and written informed consent was obtained from all participants. Because no identifying information was accessed by the investigators, Vanderbilt University’s Institutional Review Board determined that this study met the criteria for a “non-human subjects” determination.

### Dietary measurements

Data for dietary intake were collected via a 24-h dietary recall administered by a trained dietary interviewer. Total nutrient intake was calculated using the US Department of Agriculture’s survey nutrient database. To further reduce measurement error and to adjust for extraneous variation due to total energy intake, we applied the residual method described by Willett and Stampfer [21] for carbohydrate and fiber intake.

### SNP selection and genotyping

A total of 15 SNPs were considered in this analysis (Additional file 1: Table S1). The SNPs included in the study are *CDKN2A/B* (rs10811661), *NOTCH* (rs10923931), *HHEX-IDE* (rs111875), *CDC123/CAMK1D* (rs12779790), *SLC30A8* (rs13266634), *PPARG* (rs1801282), *KCNQ1* (rs2237895), *IGFBP2* (rs4402960), *ADAMTS9* (rs4607103), *CDKAL1* (rs4712523), *THADA* (rs7578597), *TCFL2* (rs7903146), *TSPAN8-LGRS* (rs7961581*), FTO* (rs8050136), and *JAZF1* (rs864745). All SNPs were previously associated with T2D (as of early 2009) in candidate gene and genome-wide association studies and were subsequently analyzed for single-SNP associations with T2D in a large meta-analysis by the PAGE study [22]. The 15 SNPs tested for gene–environment interactions were either accessed from existing data in the Genetic NHANES database or directly genotyped by EAGLE, one of the four large population-based studies of the PAGE I network, by using Sequenom or Illumina BeadXpress. In addition to genotyping experimental NHANES samples, we genotyped blinded duplicates provided by CDC and HapMap controls (*n* = 360). All EAGLE SNPs considered here were genotyped by all three NHANES studies (NHANES III, NHANES 1999–2000, and NHANES 2001–2002), had minor allele frequencies >5% in all three racial/ethnic populations, passed CDC quality control metrics, and are available for secondary analyses through NCHS/CDC.

### Statistical analysis

All analyses were limited to adults (≥18 years) and stratified by self-reported race/ethnicity to minimize possible confounding due to population stratification. Odds ratios (ORs) and 95% confidence intervals (CI) were estimated using logistic regression models with adjustment for age, sex, and BMI. The association between genotype and T2D risk was evaluated based on an additive genetic model and indexing exposure to the risk allele as reported in literature. Gene-environment interactions were modeled using a multiplicative interaction term between the environmental variable and the additively encoded SNP. All models were adjusted for the main effect of the SNP and the environmental variable, along with age, sex, kcal/day, and BMI. P values in figures and Additional file 1 are not corrected by multiple testing. However, we applied the Bonferroni correction [23] for multiple testing for single SNP analysis and for interaction analysis and if an association passed the correction we report the P value in bold.

### Genetic risk score

We calculated the GRS using all 15 SNPs. We assumed an additive genetic model for each SNP and applied a linear weighting of 0, 1, or 2 to genotypes containing 0, 1, or 2 risk alleles, respectively. Multiplicative interactions between the GRS and carbohydrate intake or fiber intake were examined by including the interactive terms in the analysis, as well as the main effects. Interaction terms were coded as the product of the GRS and the nutrient under investigation.

All analyses were performed unweighted by using SAS v9.2 (SAS Institute, Cary, NC) and the Analytic Data Research by Email (ANDRE) portal of the CDC Research Data Center in Hyattsville, MD. Data were plotted using Synthesis View [24].

## Abbreviations

BMI: Body mass index; CDC: Centers for disease control and prevention; EAGLE: Epidemiologic architecture for genes linked to environment; GRS: Genetic risk score; GWAS: Genome-wide association studies; NCHS: National center for health statistics; NHANES: National health and nutrition examination surveys; PAGE: Population architecture using genomics and epidemiology; T2D: Type 2 diabetes.

## Competing interests

The authors have no competing interests to declare.

## Authors’ contributions

RV drafted the manuscript and contributed to data analysis and interpretation. RG participated in the design of the study and performed the statistical analysis. DC conceived of the study, and participated in its design and coordination and helped to draft the manuscript. BM and JB contributed to conception and design and acquisition of data. All authors read and approved the final manuscript.

## Supplementary Material

Additional file 1: Table S1 List of SNPs. **Table S2.** Associations between GWAS SNPs and T2D*. **Table S3.** Interaction between Carbohydrates and GWAS SNPS and T2D*. **Table S4.** Interaction between Fiber and GWAS SNPS and T2D*. Click here for file
