# Supplementary material for: Gene-carbohydrate and gene-fiber interactions and type 2 diabetes in diverse populations from the National Health and Nutrition Examination Surveys (NHANES) as part of the Epidemiologic Architecture for Genes Linked to Environment (EAGLE) study
Source: BMC Genet. 2014 Jun 14;15:69. doi: 10.1186/1471-2156-15-69 (PMC4094781; doi:10.1186/1471-2156-15-69)
Supplement: Additional file 1: Table S1 — List of SNPs. Table S2. Associations between GWAS SNPs and T2D*. Table S3. Interaction between Carbohydrates and GWAS SNPS and T2D*. Table S4. Interaction between Fiber and GWAS SNPS and T2D*. [file 1471-2156-15-69-S1.docx]

|  |  | | **Table 1. List of SNPs** | | | | | | |
| --- | --- | --- | --- | --- | --- | --- | --- | --- | --- |
| **SNPs** | |  | | **Gene** | **Risk Allele** | **Other Allele** | **Non-Hispanic White RAF*** | **Non-Hispanic Black RAF*** | **Mexican American RAF*** |
| **rs10811661** | |  | | *CDKN2A/2B* | T | C | 0.82 | 0.94 | 0.87 |
| **rs10923931** | |  | | *NOTCH* | T | G | 0.10 | 0.32 | 0.09 |
| **rs1111875** | |  | | *HHEX_IDE* | G | A | 0.60 | 0.77 | 0.63 |
| **rs12779790** | |  | | *CDC123/CAMK1D* | G | A | 0.18 | 0.15 | 0.19 |
| **rs13266634** | |  | | *SLC30A8* | C | T | 0.69 | 0.90 | 0.73 |
| **rs1801282** | |  | | *PPARG* | C | G | 0.87 | 0.97 | 0.88 |
| **rs2237895** | |  | | *KCNQ1* | C | A | 0.42 | 0.18 | 0.43 |
| **rs4402960** | |  | | *IGFBP2* | T | G | 0.32 | 0.52 | 0.25 |
| **rs4607103** | |  | | *ADAMT59* | C | T | 0.74 | 0.70 | 0.68 |
| **rs4712523** | |  | | *CDKAL1* | G | A | 0.31 | 0.60 | 0.30 |
| **rs7578597** | |  | | *THADA* | T | C | 0.89 | 0.74 | 0.93 |
| **rs7903146** | |  | | *TCF7L2* | T | C | 0.29 | 0.29 | 0.21 |
| **rs7961581** | |  | | *TSPAN8-LGRS* | C | T | 0.28 | 0.21 | 0.17 |
| **rs8050136** | |  | | *FTO* | A | C | 0.40 | 0.44 | 0.25 |
| **rs864745** | |  | | *JAZF1* | A | G | 0.50 | 0.75 | 0.63 |
|  |  | | *RAF=Risk Allele Frequency | | | | | | |

|  |  | **Non-Hispanic Whites** | | | **Non-Hispanic Blacks** | | | **Mexican Americans** | | |
| --- | --- | --- | --- | --- | --- | --- | --- | --- | --- | --- |
|  |  | **Cases=545/Controls=5832** | | | **Cases=337/Controls=2717** | | | **Cases=455/Controls=3166** | | |
| **SNPs** | **Gene** | ***OR*** | ***95% C.I.*** | ***P value*** | ***OR*** | ***95% C.I.*** | ***P value*** | ***OR*** | ***95% C.I.*** | ***P value*** |
| **rs10811661** | *CDKN2A/CDKN2B* | 1.26 | (1.05,1.52) | 0.0147 | 1.23 | (0.85,1.79) | 0.2758 | 1.22 | (0.97,1.54) | 0.093 |
| **rs10923931** | *NOTCH2* | 1.21 | (0.98,1.5) | 0.0738 | 1.04 | (0.87,1.25) | 0.6773 | 1.20 | (0.94,1.55) | 0.1504 |
| **rs1111875** | *HHEX-IDE* | 1.10 | (0.96,1.26) | 0.1731 | 1.13 | (0.91,1.4) | 0.2623 | 1.15 | (0.98,1.36) | 0.0904 |
| **rs12779790** | *CAMK1D* | 1.04 | (0.88,1.24) | 0.6503 | 1.12 | (0.88,1.42) | 0.3531 | 1.03 | (0.85,1.25) | 0.7856 |
| **rs13266634** | *SLC30A8* | 1.23 | (1.06,1.42) | 0.0066 | 1.12 | (0.82,1.51) | 0.4854 | 1.25 | (1.05,1.49) | 0.0128 |
| **rs1801282** | *PPARG* | 1.02 | (0.83,1.25) | 0.8705 | 0.86 | (0.52,1.44) | 0.5735 | 0.99 | (0.78,1.26) | 0.9619 |
| **rs2237895** | *KCNQ1* | 1.08 | (0.95,1.24) | 0.2486 | 0.97 | (0.78,1.22) | 0.8032 | 1.11 | (0.95,1.29) | 0.1976 |
| **rs4402960** | *IGF2BP2* | 1.30 | (1.13,1.5) | **0.0003** | 0.87 | (0.73,1.04) | 0.1251 | 1.07 | (0.9,1.27) | 0.4187 |
| **rs4607103** | *ADAMTS9* | 0.95 | (0.82,1.11) | 0.5398 | 0.95 | (0.78,1.15) | 0.5858 | 1.07 | (0.91,1.26) | 0.4325 |
| **rs4712523** | *CDKAL1* | 1.07 | (0.92,1.23) | 0.384 | 0.88 | (0.74,1.06) | 0.1814 | 1.14 | (0.97,1.34) | 0.1135 |
| **rs7578597** | *THADA* | 1.08 | (0.87,1.36) | 0.4754 | 1.22 | (0.99,1.51) | 0.0638 | 1.04 | (0.76,1.41) | 0.8142 |
| **rs7903146** | *TCF7L2* | 1.19 | (1.03,1.38) | 0.0176 | 1.64 | (1.36,1.98) | **0.0001** | 1.33 | (1.11,1.6) | 0.0024 |
| **rs7961581** | *TSPAN8-LGR5* | 1.00 | (0.86,1.17) | 0.9737 | 0.96 | (0.78,1.19) | 0.7355 | 0.99 | (0.81,1.21) | 0.9195 |
| **rs8050136** | *FTO* | 1.04 | (0.91,1.19) | 0.5639 | 1.06 | (0.89,1.27) | 0.5308 | 1.17 | (0.99,1.39) | 0.0697 |
| **rs864745** | *JAZF1* | 1.05 | (0.92,1.2) | 0.5022 | 1.36 | (1.1,1.68) | 0.0047 | 0.96 | (0.82,1.12) | 0.5731 |

**Table 2. Associations between GWAS SNPs and T2D***

*Analysis adjusted for Age, Sex, BMI

Highlighted in bold are significance thresholds that survive a conservative Bonferrroni correction for multiple testing (p-value <0.001)

| **Table 3: Interaction between Carbohydrates and GWAS SNPS and T2D*** | | | | | | | | | | | | | | | | |
| --- | --- | --- | --- | --- | --- | --- | --- | --- | --- | --- | --- | --- | --- | --- | --- | --- |
|  |  |  |  |  |  | |  | |  | |  | |  | |  | |
|  | **Non-Hispanic Whites** | | | **Non-Hispanic Blacks** | | | | | | **Mexican American** | | | | | |  |
| ***SNPs*** | ***OR*** | ***95% C.I.*** | ***P value*** | ***OR*** | | ***95% C.I.*** | | ***P value*** | | ***OR*** | | ***95% C.I.*** | | ***P value*** | |  |
| **rs10811661** | 1.43 | (0.71,2.88) | 0.3 | 2.98 | | (0.64,13.94) | | 0.16 | | 0.6 | | (0.22,1.6) | | 0.03 | |  |
| **rs10923931** | 0.84 | (0.39,1.82) | 0.6 | 0.96 | | (0.48,1.94) | | 0.9 | | 3.06 | | (1.05,8.93) | | 0.04 | |  |
| **rs1111875** | 0.8 | (0.48,1.35) | 0.4 | 0.61 | | (0.28,1.35) | | 0.2 | | 0.85 | | (0.45,1.6) | | 0.6 | |  |
| **rs12779790** | 0.89 | (0.45,1.78) | 0.7 | 1.62 | | (0.69,3.82) | | 0.3 | | 0.48 | | (0.21,1.07) | | 0.07 | |  |
| **rs13266634** | 1.21 | (0.71,2.06) | 0.5 | 0.54 | | (0.16,1.83) | | 0.3 | | 0.55 | | (0.28,1.1) | | 0.09 | |  |
| **rs1801282** | 0.52 | (0.23,1.17) | 0.1 | 0.15 | | (0.02,1.44) | | 0.1 | | 0.75 | | (0.28,1.98) | | 0.5 | |  |
| **rs2237895** | 0.93 | (0.55,1.58) | 0.8 | 0.77 | | (0.35,1.67) | | 0.5 | | 0.93 | | (0.5,1.73) | | 0.8 | |  |
| **rs4402960** | 0.78 | (0.46,1.32) | 0.4 | 2.12 | | (1.12,4.04) | | 0.02 | | 1.05 | | (0.53,2.06) | | 0.9 | |  |
| **rs4607103** | 1.62 | (0.95,2.79) | 0.07 | 1.13 | | (0.56,2.29) | | 0.7 | | 0.79 | | (0.41,1.54) | | 0.5 | |  |
| **rs4712523** | 1.83 | (1.02,3.3) | 0.04 | 0.97 | | (0.49,1.93) | | 0.9 | | 0.85 | | (0.44,1.66) | | 0.6 | |  |
| **rs7578597** | 0.73 | (0.28,1.88) | 0.5 | 2.16 | | (1.03,4.55) | | 0.04 | | 0.58 | | (0.19,1.8) | | 0.3 | |  |
| **rs7903146** | 0.8 | (0.44,1.44) | 0.4 | 0.88 | | (0.47,1.65) | | 0.7 | | 1.34 | | (0.62,2.88) | | 0.4 | |  |
| **rs7961581** | 1.19 | (0.67,2.12) | 0.5 | 2.23 | | (0.97,5.11) | | 0.06 | | 3.21 | | (1.34,7.7) | | 0.009 | |  |
| **rs8050136** | 0.59 | (0.35,1) | 0.048 | 0.97 | | (0.49,1.92) | | 0.9 | | 1.38 | | (0.72,2.64) | | 0.3 | |  |
| **rs864745** | 1.37 | (0.8,2.36) | 0.2 | 1.21 | | (0.55,2.66) | | 0.6 | | 1.04 | | (0.55,1.97) | | 0.9 | |  |

*Adjusted for age, sex, BMI, kcal/day, SNP and carbohydrate. None of the tests for interaction survived a conservative Bonferroni correction for multiple testing.

| **Table 4: Interaction between Fiber and GWAS SNPS and T2D*** | | | | | | | | | | |
| --- | --- | --- | --- | --- | --- | --- | --- | --- | --- | --- |
|  | **Non-Hispanic Whites** | | | **Non-Hispanic Blacks** | | |  | **Mexican American** | | |
| ***SNPs*** | ***OR*** | ***95% C.I.*** | ***P value*** | ***OR*** | ***95% C.I.*** | ***P value*** |  | ***OR*** | ***95% C.I.*** | ***P value*** |
| **rs10811661** | 1.02 | (0.68,1.53) | 0.9 | 1.14 | (0.53,2.43) | 0.7 |  | 0.82 | (0.5,1.36) | 0.5 |
| **rs10923931** | 0.78 | (0.51,1.21) | 0.3 | 0.94 | (0.65,1.34) | 0.7 |  | 1.34 | (0.78,2.29) | 0.3 |
| **rs1111875** | 1.14 | (0.85,1.52) | 0.4 | 0.81 | (0.52,1.24) | 0.3 |  | 1.15 | (0.82,1.62) | 0.4 |
| **rs12779790** | 1.3 | (0.89,1.92) | 0.2 | 0.98 | (0.62,1.52) | 0.9 |  | 1.11 | (0.74,1.67) | 0.6 |
| **rs13266634** | 1.15 | (0.84,1.57) | 0.4 | 0.87 | (0.47,1.61) | 0.7 |  | 0.91 | (0.63,1.31) | 0.6 |
| **rs1801282** | 0.62 | (0.4,0.95) | 0.03 | 0.46 | (0.15,1.43) | 0.2 |  | 0.88 | (0.52,1.49) | 0.6 |
| **rs2237895** | 0.92 | (0.69,1.24) | 0.6 | 1.22 | (0.77,1.94) | 0.4 |  | 1.03 | (0.74,1.44) | 0.9 |
| **rs4402960** | 0.89 | (0.66,1.21) | 0.4 | 1.02 | (0.73,1.43) | 0.9 |  | 1.11 | (0.77,1.61) | 0.6 |
| **rs4607103** | 1.67 | (1.22,2.29) | 0.001 | 1.79 | (1.24,2.58) | 0.002 |  | 0.78 | (0.55,1.11) | 0.2 |
| **rs4712523** | 1.22 | (0.9,1.65) | 0.2 | 1.08 | (0.76,1.54) | 0.6 |  | 0.77 | (0.55,1.09) | 0.1 |
| **rs7578597** | 0.98 | (0.6,1.61) | 0.9 | 1.64 | (1.08,2.49) | 0.02 |  | 0.34 | (0.17,0.69) | 0.003 |
| **rs7903146** | 1.11 | (0.8,1.53) | 0.5 | 0.76 | (0.54,1.09) | 0.1 |  | 0.87 | (0.58,1.29) | 0.5 |
| **rs7961581** | 1 | (0.73,1.36) | 0.94 | 1.46 | (0.94,2.25) | 0.09 |  | 1.74 | (1.1,2.75) | 0.02 |
| **rs8050136** | 0.71 | (0.53,0.95) | 0.02 | 1.13 | (0.8,1.61) | 0.5 |  | 1.03 | (0.72,1.47) | 0.9 |
| **rs864745** | 1.17 | (0.87,1.57) | 0.3 | 1.04 | (0.69,1.58) | 0.8 |  | 0.77 | (0.55,1.08) | 0.1 |

*Adjusted for age, sex, BMI, kcal/day, SNP and fiber. None of the tests for interaction survived a conservative Bonferroni correction for multiple testing
